# Decision Making Strategy and the Simultaneous Processing of Syntactic Dependencies in Language and Music

**DOI:** 10.3389/fpsyg.2018.00038

**Published:** 2018-01-30

**Authors:** M. P. Roncaglia-Denissen, Fleur L. Bouwer, Henkjan Honing

**Affiliations:** ^1^Institute for Logic, Language and Computation, University of Amsterdam, Amsterdam, Netherlands; ^2^Amsterdam Brain and Cognition, University of Amsterdam, Amsterdam, Netherlands

**Keywords:** syntactic processing, language, music, local and long-distance dependencies, parser, decision making, attention

## Abstract

Despite differences in their function and domain-specific elements, syntactic processing in music and language is believed to share cognitive resources. This study aims to investigate whether the simultaneous processing of language and music share the use of a common syntactic processor or more general attentional resources. To investigate this matter we tested musicians and non-musicians using visually presented sentences and aurally presented melodies containing syntactic local and long-distance dependencies. Accuracy rates and reaction times of participants’ responses were collected. In both sentences and melodies, unexpected syntactic anomalies were introduced. This is the first study to address the processing of local and long-distance dependencies in language and music combined while reducing the effect of sensory memory. Participants were instructed to focus on language (language session), music (music session), or both (dual session). In the language session, musicians and non-musicians performed comparably in terms of accuracy rates and reaction times. As expected, groups’ differences appeared in the music session, with musicians being more accurate in their responses than non-musicians and only the latter showing an interaction between the accuracy rates for music and language syntax. In the dual session musicians were overall more accurate than non-musicians. However, both groups showed comparable behavior, by displaying an interaction between the accuracy rates for language and music syntax responses. In our study, accuracy rates seem to better capture the interaction between language and music syntax; and this interaction seems to indicate the use of distinct, however, interacting mechanisms as part of decision making strategy. This interaction seems to be subject of an increase of attentional load and domain proficiency. Our study contributes to the long-lasting debate about the commonalities between language and music by providing evidence for their interaction at a more domain-general level.

## Introduction

Language and music have many features in common. Both contain a hierarchical organization of their elements, rhythmic and melodic features and syntax (Lerdahl and Jackendoff, 1983; Patel, 2003). In the current study, we focus on music and language syntax, and more precisely on the mechanisms underlying their processing. In language and music, syntax should be understood as the rules organizing discrete elements (Patel, 2003; Arbib, 2013; Asano and Boeckx, 2015), such as words in language and notes or chords in music (Arbib, 2013). One should not, however, expect to find direct equivalence between the elements organized by language and music syntax. Nevertheless, despite the absence of direct equivalence between their constituents, language and music could still share similar underlying mechanisms organizing their elements (Patel, 2012; Peretz et al., 2015).

In Western tonal music, syntax organizes its elements by means of rhythmic and tonal stability (Asano and Boeckx, 2015), the latter being of special interest for the current work. Tonal stability should be understood in terms of tonal expectancy. That is, each key consists of seven pitches, ranging from the tonic, the most stable pitch and the tonal center of that key, to less stable pitches, like the supertonic, one step above the tonic, and the leading note, i.e., the seventh step of the scale and one step lower than the tonic. Less stable pitches are expected to resolve into more stable ones, with the tonic being the ultimate and most expected target to which all the other tones lead (Benward and Saker, 2007; Rohrmeier, 2011; Rohrmeier and Koelsch, 2012).

In language, syntax should be understood as a set of principles governing the combination of discrete and structured linguistic elements, e.g., morphemes and words, into sequences, e.g., words and sentences, respectively (Jackendoff, 2002). For instance, in an utterance in English, the use of an article will narrow down the possibilities of what the forthcoming element is, which is most likely to be a noun rather than a verb.

In terms of the relationship between syntactic processing in music and language, several different hypotheses have been proposed. First, it has been suggested that language and music make use of identical neural network in their syntactic processing (*identity hypothesis*). Alternatively, syntactic processing may occur in overlapping brain regions, sharing neural circuitry (*neural sharing hypothesis*) or it may occur in overlapping brain regions but without actively shared neural circuitry (*neural overlap hypothesis*). Finally, syntactic processing may be completely distinct from one another (*dissociation hypothesis*; please see Honing et al., 2015 for a literature review).

Among the research supporting the view of shared circuitry the idea of a common computational mechanism, i.e., the parser, is widely accepted. This is known as the Shared Syntactic Integration Resources Hypothesis (SSIRH; Patel, 1998, 2003). According to the SSIRH, the simultaneous processing of a syntactic violation in both domains (i.e., language and music) would increase the computational load for the common parser in comparison to the processing of a syntactic violation in only one of the two domains. This would impair the parser, leading to worse performances in face of a double violation (Koelsch et al., 2005; Fedorenko et al., 2009; Slevc et al., 2009).

Additionally, a facilitation effect in terms of accuracy rates and reaction times for language syntactic processing has been reported when the tonic expectation was fulfilled, supporting the SSIRH. That is, during language syntactic processing, whenever a musical tonal context was given and the tonic note occurred, better behavioral performances were reported for language syntactic processing (Hoch et al., 2011).

Despite plenty supporting experimental evidence, recent studies have challenged the view that language and music make use of shared brain areas for their syntactic processing (Fedorenko et al., 2011; Fitch and Martins, 2014) or a common syntactic parser (Perruchet and Poulin-Charronnat, 2013; Bigand et al., 2014).

It has been argued that the reported interplay between language and music syntactic processing, namely worse performance in face of a double violation, could result from shared attentional resources rather than a common parser. This should be the case because similar language and music interaction was found for the simultaneous processing of syntactic violations in music and semantic garden paths, where no language syntactic reanalysis was expected. The authors suggested that instead of shared cognitive resources in syntactic processing, shared attentional resources and cognitive control could explain the interaction between language and music syntactic processing (Perruchet and Poulin-Charronnat, 2013; Slevc and Okada, 2014).

Additionally, it has been suggested that the supposed language and music syntactic interaction could result from sensory memory effects rather than their syntactic interplay. That is, whenever an unexpected tone/chord would be heard, this created acoustic dissonance with the musical context still active in sensory memory, disrupting attention (cf. Koelsch et al., 2007; Bigand et al., 2014).

In face of the presented matters, the current research investigates whether the mechanisms underlying language and music syntactic processing are distinct or shared. In case of shared mechanisms, the current research investigates whether these would result from a common parser, shared attentional or cognitive control resources (Patel, 1998, 2003, 2008; Perruchet and Poulin-Charronnat, 2013; Slevc and Okada, 2014). Therefore, we used sentences and melodies containing local and long-distance syntactic dependencies. While in local dependencies syntactically connected elements are adjacent in the surface sequence, in long-distance dependencies these are not (Phillips et al., 2005).

In language, long-distance dependencies have been vastly investigated (Frazier, 2002) and associated with difficulty in syntactic processing due to greater working memory and attentional load (Gibson, 1998, 2000). In music, however, the use of such structure has been under-studied (cf. Patel, 2003). To our knowledge, only one study has addressed music syntactic processing using long-distance dependencies (Koelsch et al., 2013), suggesting that musicians as well as non-musicians can successfully process this kind of syntactic structure.

The simultaneous processing of long-distance dependencies in language and music, however, has not yet been investigated (Arbib, 2013). Long-distance dependencies are believed to be a syntactic feature present in language and music (Lerdahl and Jackendoff, 1983; Steedman, 1984, 1996; Rohrmeier, 2011; Rohrmeier and Koelsch, 2012).

We understand that, in terms of their specifics, such as constituents and organization rules, language and music syntax could not be perfectly mapped into each other. However, the comparison of the use of their elements during language and music simultaneous processing is still valid, as possibly common cognitive resources, such as attention (Perruchet and Poulin-Charronnat, 2013), a parser (Patel, 2003, 2008; Slevc et al., 2009) or an executive control mechanism (Slevc et al., 2013) could be involved during the syntactic processing in both domains. Thus, if language and music syntactic processing share common cognitive resources, being it a parser or more general attention or cognitive control mechanisms, these should be most visible in the processing of structures common to both domains. Understanding how language and music might interact during syntactic processing of a common feature might help to understand the mechanisms underlying these interactions.

Additionally, the use of long-distance dependencies to investigate syntactic processing in music is very suited because it helps to minimize attention disruption in face of a deviant or unexpected tone. Whenever a harmonic deviant tone occurs it creates dissonance, which conflicts with the previously heard musical context, which is still active in sensory memory (Bigand et al., 2014). However, if between the deviant tone and its linked musical context an additional music fragment is presented (as it is the case of melodies containing long-distance dependencies), and no local syntactic violation occurs, this could diminish harmonic dissonance. Less harmonic dissonance, in turn, helps to reduce attention disruption. Moreover, the higher attentional load necessary to process long-distance syntactic dependencies in comparison to the processing of local syntactic dependencies makes them more suitable to detect a possible use of shared attentional resources.

To address these matters, visual sentences and melodies containing either correct or incorrect syntactical structure in local or long-distance dependency relations were used. For the sentences, syntactic incorrectness was achieved by means of a number-agreement violation between the main and relative clause. That is, if the relative clause would not agree in number with its related noun in the main clause, this would constitute a syntactic violation. For the melodies, syntactic violations consisted of a violation in tonal expectancy. For instance, if the last tone were out of key with regard to its adjacent (in melodies containing local syntactic dependencies) and non-adjacent (melodies containing long-distance syntactic dependencies) related music context, this would constitute a syntactic violation. Syntactic violations occurred either in language, or music, in both or neither.

If cognitive resources in language and music syntactic processing are shared, being them a common parser or attention, we would expect worse performance for the double violations in comparison to a violation in one domain only. Thus, during the simultaneous processing of language and music, task disruptions in one domain could affect the other. To differentiate between the two hypotheses regarding the nature of the shared resources (i.e., a common parser or shared attention), in the current research, attention was manipulated by having participants judge the syntactic correctness of the presented stimulus material in three different session. In the language session, participants judged the syntactic correctness of language while implicitly listening to the melodies in the background. In the music session, they judged the syntactic correctness of music, while reading the visually presented sentences less attentively, as they were not task-relevant in this session. Finally, in the dual session participants were required to be prepared to judge the syntactic correctness of both language and music at all times.

With such a manipulation, if language and music syntactic processing used a common parser, worse behavioral performance would be expected in face of a double syntactic violation in all sessions, regardless of the domain being explicitly or implicitly processed. This should be the case because the parser is an automatic mechanism that operates at all times (Fodor, 1983; Kemper and Kemtes, 2002), and when required to process a violation in one domain, an additional violation in the other domain would impair its performance (Patel, 2003). However, it could be that syntactic processing in language and music becomes automatic, as of a parser, only after a certain level of proficiency is achieved. This could be the case because previous literature suggests that with proficiency cognitive processes could become more automatic (cf. Segalowitz and Hulstijn, 2005). Therefore, we tested professional musicians and non-musicians in order to address the importance of proficiency for an automatic syntactic processing in music.

If, however, the impairment of such shared resources, that is worse performance in face of the double violation, would occur only during the actively processing of both domains, this would suggest the sharing of a more general attentional mechanism and not a parser. This could be the case because when attention is divided between the processing of the two domains, less resources would be available for the processing of a double violation (Perruchet and Poulin-Charronnat, 2013).

Alternatively, syntactic processing of long-distance dependencies in language and music may not share cognitive resources, but rather depend on distinct mechanisms, which, however, could still influence one another. Whenever two distinct mechanisms are operating at the same time in response to a cognitive event, their interaction is believed to intensify the cognitive response (Hagoort, 2003). Thus, participants’ performance should be enhanced, i.e., accuracy rates increase and reaction times decrease, whenever both mechanisms are processing comparable features, i.e., syntactic violation or correctness, in a so-called syntactic congruence. Better performance in response to syntactic symmetry would then be expected in all sessions if both language and music were automatically processed.

A fourth possibility should also be contemplated, namely that distinct mechanisms are involved in processing of language and music syntactic dependencies and they do not influence each other at all. In such a case, no interaction should be found between language and music syntactic processing, not even when participants are focusing on both domains. This possibility, in our view, is the least likely to be true, as previous research report a transfer effect of expertise from one domain to the other (Jentschke and Koelsch, 2009; Moreno et al., 2009; Bhide et al., 2013; Roncaglia-Denissen et al., 2013, 2016). When a cognitive ability is affected by training and expertise in a different domain, this may be regarded as evidence for some interaction between the underlying mechanisms (Peretz et al., 2015).

## Materials and Methods

### Participants

Fifteen musicians, all actively playing during the period of data collection (5 males, *M*_age_ = 24.26, *SD* = 4.39, *M*_years of musical training_ = 15.66, *SD* = 4.06, ranging from 9 to 21 years) and 15 non-musicians (7 males, *M*_age_ = 25.66, *SD* = 3.71, *M*_years of musical training_ = 1.66, *SD* = 1.06), native speakers of Dutch, participated in three experimental sessions. In all three sessions, while the same language and music material was used, attention was differently modulated: toward language in the language session, music in the music session and divided between both, in the dual session. Participants were either university (non-musicians) or conservatory students (musicians) or had recently graduated and were paid a small fee for their participation. None of the participants reported any neurological impairment or hearing deficit and all had normal or corrected-to-normal vision. This study was approved by the ethics committee of the Faculty of Humanities of the University of Amsterdam and all participants gave their written informed consent for data collection, use, and publication.

### Material

The stimulus material consisted of 288 sentences and the same amount of melodies, and presented a 2 × 2 × 2 design, with the within-subject factors *syntactic dependency* (144 sentence containing local dependencies vs. 144 containing long-distance syntactic dependencies), *language syntax* (144 sentences presenting correct syntax vs. 144 sentence presenting incorrect syntax), and *music syntax* (144 melodies containing notes only in the correct key vs. 144 melodies containing notes in the incorrect key). This resulted in trial octuplets with each trial (sentence and melody combined) corresponding to one of the eight experimental conditions with 36 trials each: 1) *local dependency, correct language and music syntax*, 2) *local dependency, correct language and incorrect music syntax*, 3) *local dependency, incorrect language and correct music syntax*, 4) *local syntactic dependency, incorrect language and music syntax*, 5*) long-distance dependency, correct language and music syntax*, 6) *long-distance dependency, correct language and incorrect music syntax*, 7) *long-distance dependency, incorrect language and correct music syntax* and 8) *long-distance dependency, incorrect language and music syntax*. Sentences and melodies ranged from 6 to 12 words/notes long. Melodies were created in six versions to match sentences length and were transposed to all 12 keys, with each key being used 3 times across each condition. Language and music material is available as part of the Supplementary Material.

#### Language Stimulus Material

Sentences containing local syntactic dependencies consisted of one main clause in the past tense (e.g., “De buurman is vroeger bijna verdronken/*The neighbor has nearly drowned in the past”*). Long-distance syntactic dependencies were created by using a demonstrative clause in the present tense (e.g., “Daar komt de buurman/*There comes the neighbor”*) and adding a relative clause to the noun phrase (e.g., “de buurman/*the neighbor”*). The relative clause was in the past tense and followed the canonic order in Dutch, with the participle of the main verb in the second last position and the auxiliary verb in the final position (e.g., “die vroeger bijna verdronken is/*who nearly drowned in the past”*). For local syntactic dependencies, syntactic violations were created by an incorrect number agreement between the noun phrase and its verb (e.g., “De buurman_(SING)_ *zijn*_(PL)_ *vroeger bijna verdronken/ *The neighbor*_(SING)_*nearly drowned*_(PL)_*in the past*”). For long-distance dependencies, syntactic violations were created by using a verb in the relative clause which was disagreeing in number with its related noun phrase in the main clause (e.g., “Daar komt de buurman*_(SING)_ die vroeger bijna verdronken zijn_(PL)_/*There comes the neighbor*_(SING)_ *who nearly drowned*_(PL)_ *in the past”*). Conditions containing sentences with syntactically correct and incorrect long-distance dependencies differed in terms of their main clause, presenting the same relative clause.

#### Music Stimulus Material

Melodies containing local syntactic dependencies (144 tokens) were created using three parts. In the first part, a clear tonal context was set. In the second part, this context was followed by a tone that either confirmed the context or violated it. The third part was a continuation of the melody and was added to create melodies of equal length to the sentences. The first part either consisted of the key tonic followed by its dominant (e.g., C4-G4 in C Major) or two tonics interspersed by the dominant (e.g., C4-G4-C4 in C Major), setting a clear tonal context. The second part consisted of only one note, which was identical for syntactically correct and incorrect conditions. In the syntactically correct conditions this tone was a third in the key established in the first part (e.g., an E4 in the above example). To create the syntactically incorrect conditions the first part of the melody was transposed to the key 3 semitones away from the key used in the syntactically correct conditions. That is, when the first part of syntactically correct melodies was in C Major, the first part of its syntactically incorrect counterpart was in E*b* Major, making the second part (e.g., an E4) a syntactic violation. The third part was between three and seven notes long and consisted of the same notes for the syntactically correct and incorrect conditions. The third part simply continued the melody as if the second part had been a third (so in the above example, the third part would end the melody in C Major).

Similarly, melodies with long-distance dependencies contained three parts. In part one, a clear key context was established, always starting and ending with the tonic. In half of the 144 melodies, part one consisted of four tones, with the fifth and the leading note interspersed in between the tonics. In the other half, part one consisted of five tones, with one extra tonic added between the fifth and the leading note. In part two an ambiguous key context was created by using notes that fitted both in the key established in part one and in the key 3 semitones higher. For instance, if in part one the established key was C Major, the ambiguous part would present notes common to C Major and E*b* major (for example a D, F, and G). Hence, part two would conform to both, the key presented in part one (being interpreted as the second, the fourth and the fifth notes) and the key a minor third away from it (being interpreted as the leading note, the second and the third note). Part two was between four and six tones long. The third and final part consisted of one pitch only, this would either conform the key established in part one, in case of the correct music syntactic condition, or violate it, in the incorrect music syntactic condition.

**FIGURE 1:**
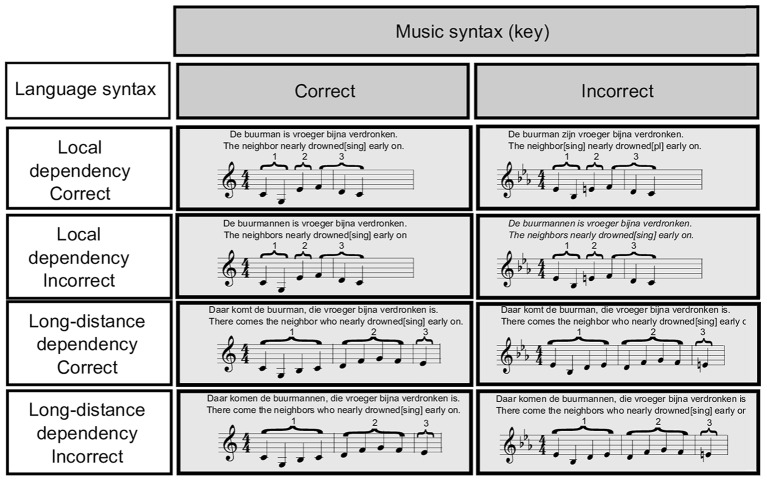
Exemplary items of the experimental material used in every condition.

The incorrect syntactic condition was created by changing the key of part one to the key a minor third higher to it, while keeping part two and three exactly the same as in the correct syntactic condition. Part three would either consist of the major third of the key used in part one (in the given example of C Major an E), while in the incorrect syntactic condition it would constitute a key violation (as in the example, an E would violate the key of E*b* Major established in part one). Thus, syntactic violation was achieved by changing the harmonic context set by part one, while keeping part two and three constant. If part three were to be interpreted as syntactically incorrect, this would have occurred because participants would have perceived it as linked to the non-adjacent context provided by part one. Melodies were created using marimba sounds (soundfont FluidR3_GM.sf2 from FluidSynth Library; GNU Lesser General Public Library^1^). Exemplary items for the eight experimental conditions are illustrated in **Figure 1**.

Based on the key fitness profile (Krumhansl and Kessler, 1982; Temperley and Marvin, 2008), key stability in the ambiguous part was calculated for both possible key interpretations: conforming part one (i.e., in the correct music syntax condition) or conforming the key three semitones higher to it (i.e., in the incorrect music syntax condition). A *t*-test revealed a significant mean difference in key stability, *t*(71) = 8.15, *p* < 0.0001, with the ambiguous part being interpreted as more stable in the correct (*M* = 17.18, *SD* = 3.11) than in the incorrect music syntactic condition (*M* = 15.95, *SD* = 4.23). This means that in the incorrect music syntactic condition the key-fitness profile of the ambiguous part would become more stable in face of a modulation to a different key than established in part one. If participants processed the syntactic dependencies as being local rather than as long-distance ones, this could lead to modulation to a more stable key, such as a key in a third down from the key in part one. In the case of such a modulation, participants would not perceive the last item in the melody as being syntactically incorrect. Thus, the mere detection of the syntactically incorrect endings in our melodies would suggest that participants processed them as containing long-distance, rather than local, syntactic dependencies.

Additionally, to account for the possibility that participants might be detecting differences between syntactically correct and incorrect melodies as a result of sensory memory priming and decay, i.e., harmonically related tones could promote a longer activation of the target tone in sensory memory, we analyzed our stimulus material using the auditory model proposed by Leman (2000). A *t*-test showed no statistically significant differences in terms of sensory memory priming between syntactically correct and incorrect melodies, *t*(1080) = -1.00, *p* = 0.31.

### Procedure

Participants were tested in a sound attenuating booth. Sentences were presented visually and melodies aurally via two loudspeakers positioned at each side of participants. Participants were tested using the same stimulus material (288 sentences and the same amount of melodies) in three different sessions. In the language session, they judged the syntactic correctness of the visually presented sentences, and in the music session, they judged the syntactic correctness of the melodies (how good the melodies sounded) while reading the visually presented sentences less attentively, as they were not task-relevant. Finally, in the dual session, participants were instructed to be prepared to judge either the syntactic correctness of sentences or melodies at all times. A response screen prompted participants to judge either language or music syntax as “correct” (visually represented on the response screen by the symbol of a happy face) or “incorrect” (visually represented on the response screen by the symbol of a sad face) at the end of each trial. Participants were instructed to respond as fast and accurate as possible regarding either language correctness or how good the melody sounded. Sessions were between two and 7 days apart from each other and their order was counterbalanced across participants.

Each sentence was presented visually in a word-by-word fashion together with a melody presented tone-by-tone, with each tone being presented in combination with a single word. Each trial began with a white fixation mark (a star) placed at the middle of a black computer screen. After 1000 ms, the fixation mark was replaced by the first word and tone. Words were presented simultaneous with tones, with inter-onset intervals of 600 ms. Trials lasted between 4.6 and 8.2 s (*M* = 6.51s*, SD* = 1.07), or 6 to 9 simultaneously presented words and tones. Immediately after the presentation of the last word and tone (with their offset), participants were presented with a response screen containing a picture of a book or a musical note, which indicated the task at hand (language or music syntactic judgment). From the presentation of this response screen onward, participant’s reaction times were collected.

In each session, the experiment was divided in 8 experimental blocks of 36 trials each and approximately 5 min long. After each block, participants were offered a break. The stimulus material was presented in a different pseudo-randomized order to each participant. After each session, participants were debriefed regarding the task they had just performed and an exit-interview was used to assess their experience during the experiment in terms of difficulty, strategy used, their perception of the stimulus material and their own performance. Additionally, participants filled out a background questionnaire to assess information about their education, language, music experience and health (Roncaglia-Denissen et al., 2016).

For data analysis, six analyses of variance (ANOVA) were computed in total. For the language and the music session separately, two ANOVAs each were computed using accuracy rates and reaction times as dependent variables. *Syntactic dependency* (local vs. long-distance syntactic dependency), *language syntax* (correct vs. incorrect) and *music syntax* (correct vs. incorrect key) were used as within-subject factors and *group* (musicians vs. non-musicians) as a between-subject factor. For the dual session, two similar ANOVAs were conducted, however, one extra within-subjects factor was added, namely *task kind (language* vs. *music syntactic judgment)*. Error rate was adjusted using Holm-Bonferroni correction.

## Results

### Accuracy Rates

#### Language Session (Judgment of Language Syntactic Correctness)

In the language session a significant interaction was found between *syntactic dependency* and *language syntax*, *F*(1,28) = 6.40, *p* = 0.017, $\eta_{p}^{2}$ = 0.18. Resolving this interaction by *syntactic dependency* a significant main effect of *language syntax* was encountered for the comprehension of local dependency only, *F*(1,28) = 12.03, *p* = 0.0017, $\eta_{p}^{2}$ = 0.27, with participants making less errors while responding to language syntactic correct (*M* = 97.68%, *SD* = 2.59) than incorrect sentences (*M* = 94.12%, *SD* = 5.85). No further significant interaction or main effects were found for accuracy rates in the language session.

#### Music Session (Judgment of Music Syntactic Correctness)

**FIGURE 2:**
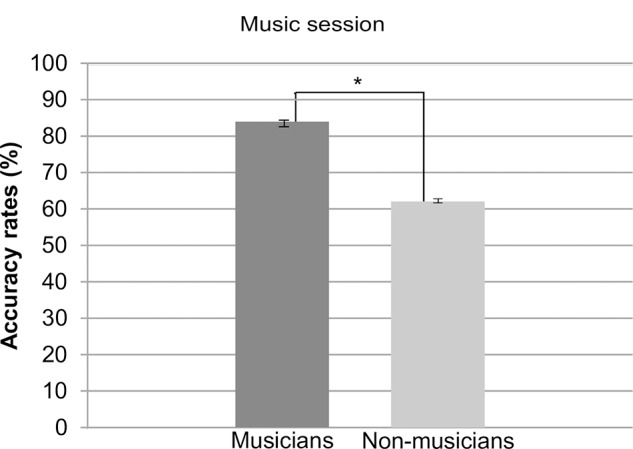
Accuracy rates for musicians and non-musicians in the music section. Error bars represent standard error.

For the music session, as expected, a main effect of *group* was found, *F*(1,28) = 26.19, *p* < 0.0001, $\eta_{p}^{2}$ = 0.48, with musicians performing overall better (*M* = 83.95% *SD* = 36.70) than non-musicians (*M* = 62.03% *SD* = 48.53). The comparison of performances of musicians and non-musicians are depicted in **Figure 2**.

**FIGURE 3:**
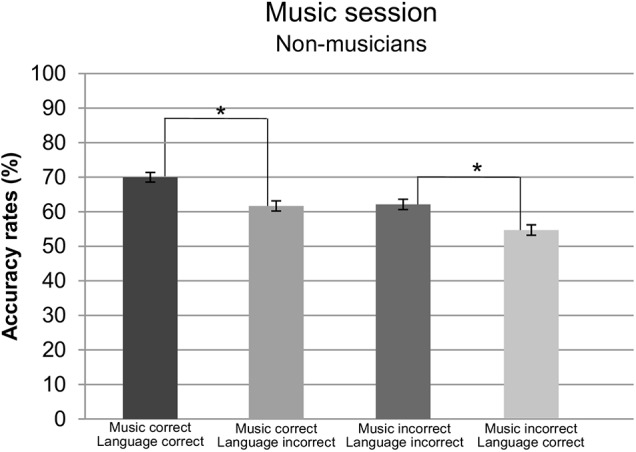
Accuracy rates for non-musicians in the music section per condition. Error bars represent standard error.

Furthermore, a two-way interaction was found for *syntactic dependency* and *group, F*(1,28) = 6.26, *p* = 0.018, $\eta_{p}^{2}$ = 0.15. Pursuing this interaction revealed a main effect of *syntactic dependency* for non-musicians only, *F*(1,14) = 26.08, *p* = 0.0002, $\eta_{p}^{2}$ = 0.65, indicating that non-musicians made less errors while responding to local dependencies (*M* = 69.30%, *SD* = 9.54) than long-distance ones (*M* = 54.76%, *SD* = 4.61). No such effect was encountered in musicians, *p* = 0.46. Finally, a significant three-way interaction was found for *group^∗^language syntax^∗^music syntax*, *F*(1,28) = 10.66, *p* = 0.0029, $\eta_{p}^{2}$ = 0.27. Resolving this interaction by *group*, an interaction between *language syntax^∗^music syntax* was found for non-musicians only, *F*(1,14) = 18.83, *p* = 0.007, $\eta_{p}^{2}$ = 0.57. Further pursue of this interaction revealed a main effect of language syntax when the melodies were syntactically correct, *F*(1,14) = 16.59, *p* = 0.001, $\eta_{p}^{2}$ = 0.64, and incorrect *F*(1,14) = 10.32, *p* = 0.0063, $\eta_{p}^{2}$ = 0.42. Interestingly enough, when melodies were syntactically correct, non-musicians made less error when implicitly reading syntactically correct (*M* = 69.85%, *SD* = 14.43) than incorrect sentences (*M* = 62.12%, *SD* = 13.03). However, when melodies were syntactically incorrect, the opposite pattern was found, namely, participants made less errors judging syntactically incorrect melodies whenever they were presented together with syntactically incorrect (*M* = 61.66%, *SD* = 9.90) than correct sentences (*M* = 54.38%, *SD* = 11.06). In both cases participants made less errors when presented with language and music syntactic congruence, i.e., when both were either correct or incorrect, than incongruence, i.e., when only one of them presented a syntactic violation. Accuracy rates for non-musicians are depicted in **Figure 3**.

#### Dual Session (Judgment of Language and Music Syntactic Correctness)

In the dual session a main effect of *group* was encountered, *F*(1,28) = 12.94, *p* = 0.0012, $\eta_{p}^{2}$ = 0.31, with musicians performing overall better (*M* = 86.50%, *SD* = 33.89) than non-musicians (*M* = 77.50%, *SD* = 41.76).

**FIGURE 4:**
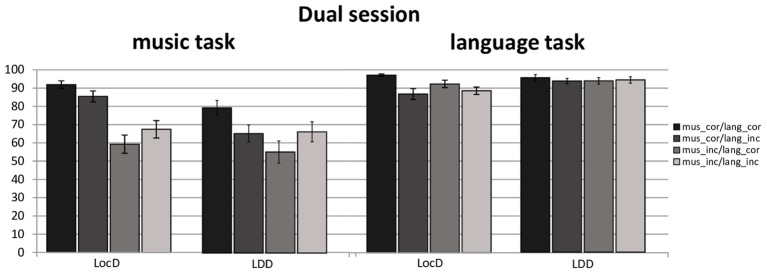
Accuracy rates for musicians and non-musicians in the dual section for the music and language task. Error bars represent standard error.

Moreover, a four-way interaction was encountered for *syntactic dependency^∗^language syntax^∗^music syntax^∗^task kind (language* vs. *music syntactic judgment)*, *F*(1,28) = 11.63, *p* = 0.0020, $\eta_{p}^{2}$ = 0.29. Further resolving this interaction by *task kind*, a three-way interaction was found for participants’ responses to music judgment, *F*(1,28) = 9.65, *p* = 0.0043, $\eta_{p}^{2}$ = 0.25. Moreover, pursuing this interaction revealed a significant two-way interaction *language syntax^∗^ music syntax*, for local, *F*(1,28) = 17.74, *p* = 0.0002, $\eta_{p}^{2}$ = 0.38, and long-distance dependencies, *F*(1,28) = 29.63, *p* < 0.0001, $\eta_{p}^{2}$ = 0.51. For local dependency, a main effect of language syntax was found for when music syntax was correct, *F*(1,28) = 11.76, *p* = 0.0019, $\eta_{p}^{2}$ = 0.29, and incorrect, *F*(1,28) = 10.99, *p* = 0.0025, $\eta_{p}^{2}$ = 0.28. For long-distance dependencies a main effect of language syntax was found for music correct, *F*(1,28) = 25.52, *p* < 0.0001, $\eta_{p}^{2}$ = 0.48, and incorrect syntax, *F*(1,28) = 10.10, *p* = 0.0036, $\eta_{p}^{2}$ = 0.26. Thus, participants made fewer errors while responding to local music and language dependencies when both were correct (*M* = 91.89%, *SD* = 11.03) and incorrect (*M* = 67.45%, *SD* = 26.05) than when music and not language was correct (*M* = 85.45%, *SD* = 16.45), or the other way around (*M* = 59.24%, *SD* = 27.33). Similar facilitation effect for syntactic congruence was encountered for long-distance dependencies. Participants made fewer response errors when either both language and music syntax were correct (*M* = 79.51%, *SD* = 22.07) or incorrect (*M* = 66.37%, *SD* = 29.67) than when only language (*M* = 65.45%, *SD* = 25.01) or music (*M* = 55.33%, *SD* = 32.75) was syntactically incorrect. Participants’ accuracy rates for the dual session are depicted in **Figure 4**.

### Reaction Times

#### Language Session (Judgment of Language Syntactic Correctness)

For the language responses a two-way interaction was found between *syntactic dependency* and *language syntax*, *F*(1,28) = 29.64, *p* < 0.0001, $\eta_{p}^{2}$ = 0.54. Resolving this interaction, we found a main effect of language syntax for local dependencies only, *F*(1,28) = 40.35, *p* < 0.0001, $\eta_{p}^{2}$ = 0.58, with participants responding faster to incorrect (*M* = 304ms, *SD* = 279) than to correct language syntax (*M* = 383ms, *SD* = 345). Such an effect could result from the tradeoff between speed and accuracy (Wickelgren, 1977; Heitz and Schall, 2012), i.e., participants were faster but also made more errors responding to language incorrect than correct syntax. No such effect was found for the process of long-distance dependencies in the language session.

#### Music Session (Judgment of Music Syntactic Correctness)

In the music session only a main effect of syntactic dependency was found, *F*(1,28) = 8.41, *p* = 0.0072, $\eta_{p}^{2}$ = 0.22, with participants responding faster to local syntactic dependencies (*M* = 426 ms, *SD* = 408) then to long-distance ones (*M* = 1004.56 ms, *SD* = 215.87). No further significant main effect and interaction were found.

#### Dual Session (Judgment of Either Language or Music Syntactic Correctness)

In the dual session, the three-way interaction *task kind (language* vs. *music syntactic judgment)^∗^syntactic dependency^∗^language syntax* was found*, F*(1,28) = 12.04, *p* = 0.0018, $\eta_{p}^{2}$ = 0.33. Resolving this interaction by *task kind*, the two-way interaction of the factors *syntactic dependency* and *language syntax* was found when participants judged the music syntax only, *F*(1,28) = 32.19, *p* < 0.0001, $\eta_{p}^{2}$ = 0.35. The follow-up analysis of this two-way interaction revealed a main effect of *syntactic dependency* when participants were presented with both correct, *F*(1,28) = 35.73, *p* < 0.0001, $\eta_{p}^{2}$ = 0.55, and incorrect language syntax, *F*(1,28) = 35.73, *p* < 0.0001, $\eta_{p}^{2}$ = 0.55. Thus, local dependencies were judged faster in both correct (*M* = 994 ms, *SD* = 533.89) and incorrect language syntax (*M* = 918 ms, *SD* = 564) than long-distance dependencies in correct (*M* = 1076.41 ms, *SD* = 525.07) and incorrect language syntax (*M* = 1181.95 ms, *SD* = 563.46). No further interactions of main effects were found in the dual session.

## Discussion

In the current study, we investigated whether processing of syntactic dependencies in language and music shares a common parser, more general attentional or cognitive control resources, or should be regarded as two distinct mechanisms. We presented musicians and non-musicians simultaneously with syntactically correct and incorrect sentences and melodies which contained local and long-distance syntactic dependencies. Participants were instructed to judge either the syntactic correctness of the sentence (language session), the syntactic correctness of the melody (music session) or to be prepared to judge the syntactic correctness of either of the two (dual session).

Similarly to previous studies, we found an interaction between syntactic processing in language and music (Fedorenko et al., 2009; Slevc et al., 2009; Hoch et al., 2011). In our study this interaction could be captured by the accuracy rates in response to the presentation of music and language syntax. However, the nature of this interaction seems to be different from what has been previously reported. It has been suggested that because language and music share a common syntactic parser for the integration of their syntactic structures, whenever a double violation occurred worse performance would be predicted. Language and music reanalysis would be, therefore, impaired due to their shared parser (Patel, 1998, 2003).

Instead of the predicted difficulty increase for the double violation, we found a facilitation effect in face of a syntactic congruence (i.e., when language and music syntax were either correct or incorrect). Moreover, this effect was encountered for non-musicians, judging music syntax, i.e., music session, and both musicians and non-musicians in the dual session, i.e., when participants would not know whether they would be judging language or music syntactic correctness until at the end of each trial and should be prepared to judge syntactic correctness in both domains at all times. Perhaps, the group difference found in the music session could rely partly on musicians’ superior musical expertise and partly in their superior executive control, as a result of the cognitive demands of their musical training (Brandler and Rammsayer, 2003; Bialystok and DePape, 2009; Pallesen et al., 2010; Williamson et al., 2010; Zioga et al., 2016). Therefore, musicians might be better able to direct their attention to the task-relevant cues in the perception of music syntax (Paas and Merriënboer, 1994).

Furthermore, the similar performance between non-musicians and musicians in the dual session might result from its high cognitive demand. Perhaps, whenever the cognitive load becomes too high, individuals behave similarly, regardless of their previous knowledge and expertise on that matter (Paas and Merriënboer, 1994), relying on more domain general cognitive mechanisms.

The facilitation effect in face of syntactic congruence might suggest that during the simultaneous processing of language and music syntax distinct mechanisms operate and interact with each other. It has been suggested that whenever the same mechanism is used in the processing of distinct cognitive events, e.g., language and music syntax, cognitive resources of this mechanism would be split between the two events, yielding to worse processing performance. In contrast, whenever two distinct but interacting mechanisms are enrolled in the processing of different cognitive events, a facilitation effect may occur, creating an additive effect, e.g., higher accuracy rates and faster reaction times (Besson et al., 1992; Roberts and Sternberg, 1993; Hagoort, 2003; Sternberg, 2013), in line with our findings.

One possible explanation for the interaction of these two distinct mechanisms could be that both are related to an action executing plan to achieve a certain goal (Asano and Boeckx, 2015), such as decision making. As previous literature suggests, a decision is reached as soon as enough perceptual cues are gathered to commit to it (Heekeren et al., 2008). This is known as sequential analysis process (Gold and Shadlen, 2007; Yang and Shadlen, 2007; Pleskac and Busemeyer, 2010). As soon as enough cues are provided for a decision to be reached, a decision will be made, so resources can be freed to be recruited and used by further cognitive processing. If two different mechanisms operate in language and music syntactic processing, it may be that both are gathering information to reach a decision about the syntactic nature of the perceived input. The more information about the input the faster and the more accurately the threshold for decision making can be reached (Gold and Shadlen, 2007). This could explain the better performance in the processing of syntactic congruence (i.e., both music and language being either syntactically correct or incorrect), in comparison with the incongruence (i.e., syntactic incorrectness in only one domain).

Additionally, contrary to what would be expected if language and music shared a syntactic parser, their interaction did not occur in all tasks, but only when non-musicians were instructed to judge music syntax in the music session, and when both groups performed in the dual session. As a parser is an automatic mechanism (Fodor, 1983; Kemper and Kemtes, 2002), present at all times, its presence does not seem to explain our findings.

The suppression of the non-relevant syntactic material (language in the music session and music in the language session) seems to be subject to executive control and proficiency. Thus, whenever proficiency was kept constant (in the language session, for instance), musicians and non-musicians performed similarly. However, when proficiency differed (in the music session), musicians were better able to suppress language while non-musicians were not. Finally, in the dual session, when attention load was very high, both groups seemed to be distracted by the asymmetric syntactic pattern. This would parallel with previous research reporting worse behavioral performance of individuals when attentional load was too high (Kane and Engle, 2000; Unsworth and Engle, 2007).

Previously, it has been reported that musical syntax was pre-attentively processed during language syntactic processing (Koelsch et al., 2005; Fedorenko et al., 2009; Slevc et al., 2009; Perruchet and Poulin-Charronnat, 2013). However, these studies, differently from ours, did not control for the effect of acoustic dissonance of an unexpected or deviant sounds or sensory memory priming. Thus, one cannot rule out that the interaction found between music and language could be explained by attention disruption rather than by syntactic processing (Bigand et al., 2014).

In face of the presented results, some issues still remain. One could argue that the long-distance dependencies in the melodies were in fact processed rather locally by our participants. However, due to the nature of our stimulus material, this does not seem to be the case. In the incorrect syntactic condition, if the syntactically ambiguous part were interpreted locally, this would lead to a modulation to a different key from the one established in the beginning of the melody, due to the stronger stability that such a modulation would create. Therefore, if melodies would be locally processed, participants would not have perceived the final item in the incorrect melodies as being syntactically violated, as it was not a violation of the local context. Our results show that participants did perceive the incorrect melodies as containing a syntactic violation, suggesting that they were linking the final item to the first given musical context and not to the adjacent syntactically ambiguous one. Thus, long-distance dependencies were perceived as such in the melodies.

Similarly to what previous literature has encountered for language syntactic processing, our results reveal that local dependency are processed more accurately than long-distance ones (Gibson, 1998, 2000). In addition, in the music session similar effect was observed for non-musicians only, while no difference was encountered for musicians. This group difference could be explained by a possible increase of cognitive control ability among musicians as a result of their high proficiency in the music domain (cf. Segalowitz and Hulstijn, 2005). However, to further account for such a possibility, further studies investigating the role of proficiency in cognitive control ability among musicians are needed.

Additionally, while our findings suggest different mechanisms for the processing of syntactic dependencies in language and music, the measures here collected (i.e., accuracy rates and reaction times) are offline measures. Namely, whenever a decision toward the stimulus material is made, many cognitive processes have already taken place prior to it. This, together with the near ceiling effect in participants’ performance in judging language syntactic correctness in the language session, could be explain the lack of interaction between accuracy rates for language and music syntax. Music and language could still share the same syntactic parser, but the tools used in the current study, i.e., accuracy rates and reaction times, combined, perhaps, with the ease of the language task, might not have captured it.

Similar reasoning can be drawn for the non-automatic processing of music. It could be that with more sensitive online measures the unfolding nature or music syntactic processing could be captured and components of an automatic syntactic processing may then be observed. Further research is therefore needed to shed more light on this matter. The use of online methods, such as ERPs would help to reveal more about the nature of the cognitive processes of syntax processing in language and music, when attention presents an important element.

## Conclusion

Our study was the first to study the simultaneous syntactic processing of local and long-distance dependencies in language and music. Our findings suggest that syntactic processing in music and language relies on distinct mechanisms that may interact at the processing stage of decision making. Such a shared decision making strategy could either explain what previous research reported as shared resources between language and music processing. Future research using online methods should be conducted, in order to further elucidate this matter.

## Author Contributions

MPR-D: contributed in conceptualization, experimental design, stimulus creation, data collection, and manuscript writing. FB: contributed in stimulus creation and manuscript writing. HH: contributed in supervision.

## Conflict of Interest Statement

The authors declare that the research was conducted in the absence of any commercial or financial relationships that could be construed as a potential conflict of interest.

## Footnotes

1 www.sourceforge.net/projects/fluidsynth
