# Supplementary material for: Decision Making Strategy and the Simultaneous Processing of Syntactic Dependencies in Language and Music
Source: Front Psychol. 2018 Jan 30;9:38. doi: 10.3389/fpsyg.2018.00038 (PMC5797648; doi:10.3389/fpsyg.2018.00038)
Supplement: Supplementary file 5 [file Data_Sheet_1.PDF]

Supplementary material

Accuracy rates

Non-musicians - Language task							
**Local dependencies**							
Lang-cor/music-cor		Lang-cor/music-inc		Lang-inc/music-cor		Lang-inc/music-inc	
Mean (%)	*SD*	*Mean (%)*	*SD*	*Mean (%)*	*SD*	*Mean (%)*	*SD*
97,96	14,14	97,58	15,35	93,14	25,28	94,26	23,26
**Long-distance dependencies**							
Lang-cor/music-cor		Lang-cor/music-inc		Lang-inc/music-cor		Lang-inc/music-inc	
Mean (%)	*SD*	*Mean (%)*	*SD*	*Mean (%)*	*SD*	*Mean (%)*	*SD*
98,14	13,49	97,96	14,13	96,84	17,49	97,22	16,43

Table 1: Mean accuracy rates and standard deviations of non-musicians for all conditions in the language task.

Musicians - Language task							
**Local dependencies**							
Lang-cor/music-cor		Lang-cor/music-inc		Lang-inc/music-cor		Lang-inc/music-inc	
Mean (%)	*SD*	*Mean (%)*	*SD*	*Mean (%)*	*SD*	*Mean (%)*	*SD*
97,78	14,73	97,41	15,90	95,00	21,80	94,07	23,62
**Long-distance dependencies**							
Lang-cor/music-cor		Lang-cor/music-inc		Lang-inc/music-cor		Lang-inc/music-inc	
Mean (%)	*SD*	*Mean (%)*	*SD*	*Mean (%)*	*SD*	*Mean (%)*	*SD*
96,47	18,45	96,12	19,32	97,59	15,34	97,40	15,91

Table 2: Mean accuracy rates and standard deviations of musicians for all conditions in the language task.

Non-musicians - Music task							
Local dependencies							
Lang-cor/music-cor		Lang-cor/music-inc		Lang-inc/music-cor		Lang-inc/music-inc	
Mean (%)	*SD*	*Mean (%)*	*SD*	*Mean (%)*	*SD*	*Mean (%)*	*SD*
83,49	37,16	53,97	49,89	75,19	43,23	64,63	47,86
Long-distance dependencies							
Lang-cor/music-cor		Lang-cor/music-inc		Lang-inc/music-cor		Lang-inc/music-inc	
Mean (%)	*SD*	*Mean (%)*	*SD*	*Mean (%)*	*SD*	*Mean (%)*	*SD*
56,48	49,62	54,81	49,81	49,07	50,04	58,70	49,28

Table 3: Accuracy rates and standard deviations of non-musicians for all conditions in the music task.

Musicians - Music task							
Local dependencies							
Lang-cor/music-cor		Lang-cor/music-inc		Lang-inc/music-cor		Lang-inc/music-inc	
Mean (%)	*SD*	*Mean (%)*	*SD*	*Mean (%)*	*SD*	*Mean (%)*	*SD*
96,48	18,43	71,48	45,17	97,41	15,90	76,11	42,66
Long-distance dependencies							
Lang-cor/music-cor		Lang-cor/music-inc		Lang-inc/music-cor		Lang-inc/music-inc	
Mean (%)	*SD*	*Mean (%)*	*SD*	*Mean (%)*	*SD*	*Mean (%)*	*SD*
79,26	40,56	84,63	36,08	80,37	39,74	85,93	34,79

Table 4: Accuracy rates and standard deviations of musicians for conditions in the music task.

Non-musicians - Dual task							
Local dependencies							
Lang-cor/music-cor		Lang-cor/music-inc		Lang-inc/music-cor		Lang-inc/music-inc	
Mean (%)	*SD*	*Mean (%)*	*SD*	*Mean (%)*	*SD*	*Mean (%)*	*SD*
91,11	28,48	70,93	45,45	81,30	39,03	72,78	44,55
Long-distance dependencies							
Lang-cor/music-cor		Lang-cor/music-inc		Lang-inc/music-cor		Lang-inc/music-inc	
Mean (%)	*SD*	*Mean (%)*	*SD*	*Mean (%)*	*SD*	*Mean (%)*	*SD*
84,63	36,10	67,96	46,71	76,85	42,22	74,44	43,66

Table 5: Mean accuracy rates and standard deviations of non-musicians for all conditions in the dual task.

Musicians - Dual task							
Local dependencies							
Lang-cor/music-cor		Lang-cor/music-inc		Lang-inc/music-cor		Lang-inc/music-inc	
Mean (%)	*SD*	*Mean (%)*	*SD*	*Mean (%)*	*SD*	*Mean (%)*	*SD*
98,15	13,49	81,11	39,16	90,93	28,74	83,52	37,12
Long-distance dependencies							
Lang-cor/music-cor		Lang-cor/music-inc		Lang-inc/music-cor		Lang-inc/music-inc	
Mean (%)	*SD*	*Mean (%)*	*SD*	*Mean (%)*	*SD*	*Mean (%)*	*SD*
90,37	*29,51*	*81,11*	*39,16*	*82,78*	*37,77*	*86,11*	*34,60*

Table 6: Mean accuracy rates and standard deviations of musicians for all conditions in the dual task.

Reaction times

Non-musicians - Language task							
Local dependencies							
Lang-cor/music-cor		Lang-cor/music-inc		Lang-inc/music-cor		Lang-inc/music-inc	
Mean (ms)	*SD*	*Mean (ms)*	*SD*	*Mean (ms)*	*SD*	*Mean (ms)*	*SD*
4068.71	3918.69	3689.63	3388.72	3274.88	3377.68	2954.55	2497.20
Long-distance dependencies							
Lang-cor/music-cor		Lang-cor/music-inc		Lang-inc/music-cor		Lang-inc/music-inc	
Mean (ms)	*SD*	*Mean (ms)*	*SD*	*Mean (ms)*	*SD*	*Mean (ms)*	*SD*
3244.19	3457.22	3269.73	3572.16	3489.25	3374.54	3568.76	3543.43

Table 7: Mean reaction times and standard deviations of non-musicians for all conditions in the language task.

Musicians - Language task							
Local dependencies							
Lang-cor/music-cor		Lang-cor/music-inc		Lang-inc/music-cor		Lang-inc/music-inc	
Mean (ms)	*SD*	*Mean (ms)*	*SD*	*Mean (ms)*	*SD*	*Mean (ms)*	*SD*
3693.70	3142.92	3921.65	3521.75	2965.51	2686.50	3058.67	2708.26
Long-distance dependencies							
Lang-cor/music-cor		Lang-cor/music-inc		Lang-inc/music-cor		Lang-inc/music-inc	
Mean (ms)	*SD*	*Mean (ms)*	*SD*	*Mean (ms)*	*SD*	*Mean (ms)*	*SD*
3149.58	3097.65	3124.79	2558.54	3096.55	2908.58	3039.62	2720.14

Table 8: Mean reaction times and standard deviations of musicians for all conditions in the language task.

Non-musicians - Music task							
Local dependencies							
Lang-cor/music-cor		Lang-cor/music-inc		Lang-inc/music-cor		Lang-inc/music-inc	
Mean (ms)	*SD*	*Mean (ms)*	*SD*	*Mean (ms)*	*SD*	*Mean (ms)*	*SD*
5146.10	4434.16	4885.78	4427.71	5248.89	4665.35	4683.06	4001.30
Long-distance dependencies							
Lang-cor/music-cor		Lang-cor/music-inc		Lang-inc/music-cor		Lang-inc/music-inc	
Mean (ms)	*SD*	*Mean (ms)*	*SD*	*Mean (ms)*	*SD*	*Mean (ms)*	*SD*
6200.26	4809.00	5487.93	4395.80	6398.80	4553.28	5975.36	5509.15

Table 9: Mean reaction times and standard deviations of non-musicians for all conditions in the music task.

Musicians - Music task							
Local dependencies							
Lang-cor/music-cor		Lang-cor/music-inc		Lang-inc/music-cor		Lang-inc/music-inc	
Mean (ms)	*SD*	*Mean (ms)*	*SD*	*Mean (ms)*	*SD*	*Mean (ms)*	*SD*
3485.15	2862.48	4674.32	5138.00	3676.67	3542.67	4257.99	4008.82
Long-distance dependencies							
Lang-cor/music-cor		Lang-cor/music-inc		Lang-inc/music-cor		Lang-inc/music-inc	
Mean (ms)	*SD*	*Mean (ms)*	*SD*	*Mean (ms)*	*SD*	*Mean (ms)*	*SD*
5177.14	5494.04	4185.07	4236.68	5516.71	6176.85	4706.92	5191.20

Table 10: Mean reaction times and standard deviations of musicians for all conditions in the music task.

Non-musicians - Dual task							
Local dependencies							
Lang-cor/music-cor		Lang-cor/music-inc		Lang-inc/music-cor		Lang-inc/music-inc	
Mean (ms)	*SD*	*Mean (ms)*	*SD*	*Mean (ms)*	*SD*	*Mean (ms)*	*SD*
9901.86	5619.12	9191.36	4330.42	9018.05	5116.65	8305.30	5559.50
Long-distance dependencies							
Lang-cor/music-cor		Lang-cor/music-inc		Lang-inc/music-cor		Lang-inc/music-inc	
Mean (ms)	*SD*	*Mean (ms)*	*SD*	*Mean (ms)*	*SD*	*Mean (ms)*	*SD*
9663.81	5395.62	9631.94	5491.40	9869.81	5340.90	9790.77	6121.69

Table 11: Mean reaction times and standard deviations of non-musicians for all conditions in the dual task.

Musicians - Dual task							
Local dependencies							
Lang-cor/music-cor		Lang-cor/music-inc		Lang-inc/music-cor		Lang-inc/music-inc	
Mean (ms)	*SD*	*Mean (ms)*	*SD*	*Mean (ms)*	*SD*	*Mean (ms)*	*SD*
9087.22	4739.14	10866.80	5509.02	9186.79	4595.15	8737.43	5593.47
Long-distance dependencies							
Lang-cor/music-cor		Lang-cor/music-inc		Lang-inc/music-cor		Lang-inc/music-inc	
Mean (ms)	*SD*	*Mean (ms)*	*SD*	*Mean (ms)*	*SD*	*Mean (ms)*	*SD*
9680.04	4860.97	10860.67	5166.38	10398.17	5492.49	9483.34	4951.53

Table 12: Mean reaction times and standard deviations of musicians for all conditions in the dual task.
