# Supplementary material for: Decision Making Strategy and the Simultaneous Processing of Syntactic Dependencies in Language and Music
Source: Front Psychol. 2018 Jan 30;9:38. doi: 10.3389/fpsyg.2018.00038 (PMC5797648; doi:10.3389/fpsyg.2018.00038)
Supplement: Supplementary file 6 [file Data_Sheet_2.PDF]

| | Supplementary material: language stimulus material | Condition |
|---|---|---|
| 1 | Daar komen de buurmannen die vroeger bijna verdronken zijn. | LDD_LCMC |
| 2 | Daar komt de buurman die vroeger bijna verdronken is. | LDD_LCMI |
| 3 | Daar komen de buurmannen die vroeger bijna verdronken is. | LDD_LIMC |
| 4 | Daar komt de buurman die vroeger bijna verdronken zijn. | LDD_LIMI |
| 5 | De buurmannen zijn vroeger bijna verdronken. | LcD_LCMC |
| 6 | De buurman is vroeger bijna verdronken. | LcD_LCMI |
| 7 | De buurmannen is vroeger bijna verdronken. | LcD_LIMC |
| 8 | De buurman zijn vroeger bijna verdronken. | LcD_LIMI |
| 9 | Daar staat de jonge vrouw die na het drukke feest afgedropen is. | LDD_LCMC |
| 10 | Daar staan de jonge vrouwen die na het drukke feest afgedropen zijn. | LDD_LCMI |
| 11 | Daar staat de jonge vrouw die na het drukke feest afgedropen zijn. | LDD_LIMC |
| 12 | Daar staan de jonge vrouwen die na het drukke feest afgedropen is. | LDD_LIMI |
| 13 | De jonge vrouw is na het drukke feest afgedropen. | LcD_LCMC |
| 14 | De jonge vrouwen zijn na het drukke feest afgedropen. | LcD_LCMI |
| 15 | De jonge vrouw zijn na het drukke feest afgedropen. | LcD_LIMC |
| 16 | De jonge vrouwen is na het drukke feest afgedropen. | LcD_LIMI |
| 17 | Daar komen de rebelse dochters die in de oude woning binnengedrongen zijn. | LDD_LCMC |
| 18 | Daar komt de rebelse dochter die in de oude woning binnengedrongen is. | LDD_LCMI |
| 19 | Daar komen de rebelse dochters die in de oude woning binnengedrongen is. | LDD_LIMC |
| 20 | Daar komt de rebelse dochter die in de oude woning binnengedrongen zijn. | LDD_LIMI |
| 21 | De rebelse dochters zijn in de oude woning binnengedrongen. | LcD_LCMC |
| 22 | De rebelse dochter is in de oude woning binnengedrongen. | LcD_LCMI |
| 23 | De rebelse dochters is in de oude woning binnengedrongen. | LcD_LIMC |
| 24 | De rebelse dochter zijn in de oude woning binnengedrongen. | LcD_LIMI |
| 25 | Daar staat de leerlinge die door het moeilijke voorval geschrokken is. | LDD_LCMC |
| 26 | Daar staan de leerlingen die door het moeilijke voorval geschrokken zijn. | LDD_LCMI |
| 27 | Daar staat de leerlinge die door het moeilijke voorval geschrokken zijn. | LDD_LIMC |
| 28 | Daar staan de leerlingen die door het moeilijke voorval geschrokken is. | LDD_LIMI |
| 29 | De leerlinge is door het moeilijke voorval geschrokken. | LcD_LCMC |
| 30 | De leerlingen zijn door het moeilijke voorval geschrokken. | LcD_LCMI |
| 31 | De leerlinge zijn door het moeilijke voorval geschrokken. | LcD_LIMC |
| 32 | De leerlingen is door het moeilijke voorval geschrokken. | LcD_LIMI |
| 33 | Daar komen de sportieve meneren die naar het bos gewandeld zijn. | LDD_LCMC |
| 34 | Daar komt de sportieve meneer die naar het bos gewandeld is. | LDD_LCMI |
| 35 | Daar komen de sportieve meneren die naar het bos gewandeld is. | LDD_LIMC |
| 36 | Daar komt de sportieve meneer die naar het bos gewandeld zijn. | LDD_LIMI |
| 37 | De sportieve meneren zijn naar het bos gewandeld. | LcD_LCMC |
| 38 | De sportieve meneer is naar het bos gewandeld. | LcD_LCMI |

| | | |
|---|---|---|
| 39 | De sportieve meneren is naar het bos gewandeld. | LcD_LIMC |
| 40 | De sportieve meneer zijn naar het bos gewandeld. | LcD_LIMI |
| 41 | Daar staat de buurvrouw die sinds afgelopen zomer gescheiden is. | LDD_LCMC |
| 42 | Daar staan de buurvrouwen die sinds afgelopen zomer gescheiden zijn. | LDD_LCMI |
| 43 | Daar staat de buurvrouw die sinds afgelopen zomer gescheiden zijn. | LDD_LIMC |
| 44 | Daar staan de buurvrouwen die sinds afgelopen zomer gescheiden is. | LDD_LIMI |
| 45 | De buurvrouw is sinds afgelopen zomer gescheiden. | LcD_LCMC |
| 46 | De buurvrouwen zijn sinds afgelopen zomer gescheiden. | LcD_LCMI |
| 47 | De buurvrouw zijn sinds afgelopen zomer gescheiden. | LcD_LIMC |
| 48 | De buurvrouwen is sinds afgelopen zomer gescheiden. | LcD_LIMI |
| 49 | Daar komen de kokkinnen die door Frankrijk getrokken zijn. | LDD_LCMC |
| 50 | Daar komt de kokkin die door Frankrijk getrokken is. | LDD_LCMI |
| 51 | Daar komen de kokkinnen die door Frankrijk getrokken is. | LDD_LIMC |
| 52 | Daar komt de kokkin die door Frankrijk getrokken zijn. | LDD_LIMI |
| 53 | De kokkinnen zijn door Frankrijk getrokken. | LcD_LCMC |
| 54 | De kokkin is door Frankrijk getrokken. | LcD_LCMI |
| 55 | De kokkinnen is door Frankrijk getrokken. | LcD_LIMC |
| 56 | De kokkin zijn door Frankrijk getrokken. | LcD_LIMI |
| 57 | Daar staat de collega die op de vloer flauwgevallen is. | LDD_LCMC |
| 58 | Daar staan de collega's die op de vloer flauwgevallen zijn. | LDD_LCMI |
| 59 | Daar staat de collega die op de vloer flauwgevallen zijn. | LDD_LIMC |
| 60 | Daar staan de collega's die op de vloer flauwgevallen is. | LDD_LIMI |
| 61 | De collega is op de vloer flauwgevallen. | LcD_LCMC |
| 62 | De collega's zijn op de vloer flauwgevallen. | LcD_LCMI |
| 63 | De collega zijn op de vloer flauwgevallen. | LcD_LIMC |
| 64 | De collega's is op de vloer flauwgevallen. | LcD_LIMI |
| 65 | Daar komen de belangrijke bezoekers die sinds de laatste afspraak verdwenen zijn. | LDD_LCMC |
| 66 | Daar komt de belangrijke bezoeker die sinds de laatste afspraak verdwenen is. | LDD_LCMI |
| 67 | Daar komen de belangrijke bezoekers die sinds de laatste afspraak verdwenen is. | LDD_LIMC |
| 68 | Daar komt de belangrijke bezoeker die sinds de laatste afspraak verdwenen zijn. | LDD_LIMI |
| 69 | De belangrijke bezoekers zijn sinds de laatste afspraak verdwenen. | LcD_LCMC |
| 70 | De belangrijke bezoeker is sinds de laatste afspraak verdwenen. | LcD_LCMI |
| 71 | De belangrijke bezoekers is sinds de laatste afspraak verdwenen. | LcD_LIMC |
| 72 | De belangrijke bezoeker zijn sinds de laatste afspraak verdwenen. | LcD_LIMI |
| 73 | Daar staat de kleine jongen die onder de nieuwe tafel gekropen is. | LDD_LCMC |
| 74 | Daar staan de kleine jongens die onder de nieuwe tafel gekropen zijn. | LDD_LCMI |
| 75 | Daar staat de kleine jongen die onder de nieuwe tafel gekropen zijn. | LDD_LIMC |
| 76 | Daar staan de kleine jongens die onder de nieuwe tafel gekropen is. | LDD_LIMI |
| 77 | De kleine jongen is onder de nieuwe tafel gekropen. | LcD_LCMC |
| 78 | De kleine jongens zijn onder de nieuwe tafel gekropen. | LcD_LCMI |

| | | |
|---|---|---|
| 79 | De kleine jongen zijn onder de nieuwe tafel gekropen. | LcD_LIMC |
| 80 | De kleine jongens is onder de nieuwe tafel gekropen. | LcD_LIMI |
| 81 | Daar komen de gelukkige actrices die snel beroemd geworden zijn. | LDD_LCMC |
| 82 | Daar komt de gelukkige actrice die snel beroemd geworden is. | LDD_LCMI |
| 83 | Daar komen de gelukkige actrices die snel beroemd geworden is. | LDD_LIMC |
| 84 | Daar komt de gelukkige actrice die snel beroemd geworden zijn. | LDD_LIMI |
| 85 | De gelukkige actrices zijn snel beroemd geworden. | LcD_LCMC |
| 86 | De gelukkige actrice is snel beroemd geworden. | LcD_LCMI |
| 87 | De gelukkige actrices is snel beroemd geworden. | LcD_LIMC |
| 88 | De gelukkige actrice zijn snel beroemd geworden. | LcD_LIMI |
| 89 | Daar staat de docent die de grote toets vergeten is. | LDD_LCMC |
| 90 | Daar staan de docenten die de grote toets vergeten zijn. | LDD_LCMI |
| 91 | Daar staat de docent die de grote toets vergeten zijn. | LDD_LIMC |
| 92 | Daar staan de docenten die de grote toets vergeten is. | LDD_LIMI |
| 93 | De docent is de grote toets vergeten. | LcD_LCMC |
| 94 | De docenten zijn de grote toets vergeten. | LcD_LCMI |
| 95 | De docent zijn de grote toets vergeten. | LcD_LIMC |
| 96 | De docenten is de grote toets vergeten. | LcD_LIMI |
| 97 | Daar komen de ervaren chauffeurs die voor de gehuurde vrachtwagen geweken zijn. | LDD_LCMC |
| 98 | Daar komt de ervaren chauffeur die voor de gehuurde vrachtwagen geweken is. | LDD_LCMI |
| 99 | Daar komen de ervaren chauffeurs die voor de gehuurde vrachtwagen geweken is. | LDD_LIMC |
| 100 | Daar komt de ervaren chauffeur die voor de gehuurde vrachtwagen geweken zijn. | LDD_LIMI |
| 101 | De ervaren chauffeurs zijn voor de gehuurde vrachtwagen geweken. | LcD_LCMC |
| 102 | De ervaren chauffeur is voor de gehuurde vrachtwagen geweken. | LcD_LCMI |
| 103 | De ervaren chauffeurs is voor de gehuurde vrachtwagen geweken. | LcD_LIMC |
| 104 | De ervaren chauffeur zijn voor de gehuurde vrachtwagen geweken. | LcD_LIMI |
| 105 | Daar saat de aannemer die zeer verantwoordelijk gebleken is. | LDD_LCMC |
| 106 | Daar staan de aannemers die zeer verantwoordelijk gebleken zijn. | LDD_LCMI |
| 107 | Daar staat de aannemer die zeer verantwoordelijk gebleken zijn. | LDD_LIMC |
| 108 | Daar staan de aannemers die zeer verantwoordelijk gebleken is. | LDD_LIMI |
| 109 | De aannemer is zeer verantwoordelijk gebleken. | LcD_LCMC |
| 110 | De aannemers zijn zeer verantwoordelijk gebleken. | LcD_LCMI |
| 111 | De aannemer zijn zeer verantwoordelijk gebleken. | LcD_LIMC |
| 112 | De aannemers is zeer verantwoordelijk gebleken. | LcD_LIMI |
| 113 | Daar komen de gespierde atleten die als eerste geëindigd zijn. | LDD_LCMC |
| 114 | Daar komt de gespierde atlete die als eerste geëindigd is. | LDD_LCMI |
| 115 | Daar komen de gespierde atleten die als eerste geëindigd is. | LDD_LIMC |
| 116 | Daar komt de gespierde atlete die als eerste geëindigd zijn. | LDD_LIMI |
| 117 | De gespierde atleten zijn als eerste geëindigd. | LcD_LCMC |
| 118 | De gespierde atlete is als eerste geëindigd. | LcD_LCMI |

| | | |
|---|---|---|
| 119 | De gespierde atleten is als eerste geëindigd. | LcD_LIMC |
| 120 | De gespierde atlete zijn als eerste geëindigd. | LcD_LIMI |
| 121 | Daar staat de beroemde dichter die in aanzien gestegen is. | LDD_LCMC |
| 122 | Daar staan de beroemde dichters die in aanzien gestegen zijn. | LDD_LCMI |
| 123 | Daar staat de beroemde dichter die in aanzien gestegen zijn. | LDD_LIMC |
| 124 | Daar staan de beroemde dichters die in aanzien gestegen is. | LDD_LIMI |
| 125 | De beroemde dichter is in aanzien gestegen. | LcD_LCMC |
| 126 | De beroemde dichters zijn in aanzien gestegen. | LcD_LCMI |
| 127 | De beroemde dichter zijn in aanzien gestegen. | LcD_LIMC |
| 128 | De beroemde dichters is in aanzien gestegen. | LcD_LIMI |
| 129 | Daar komen de verkoopsters die vanmorgen al heel vroeg opgestaan zijn. | LDD_LCMC |
| 130 | Daar komt de verkoopster die vanmorgen al heel vroeg opgestaan is. | LDD_LCMI |
| 131 | Daar komen de verkoopsters die vanmorgen al heel vroeg opgestaan is. | LDD_LIMC |
| 132 | Daar komt de verkoopster die vanmorgen al heel vroeg opgestaan zijn. | LDD_LIMI |
| 133 | De verkoopsters zijn vanmorgen al heel vroeg opgestaan. | LcD_LCMC |
| 134 | De verkoopster is vanmorgen al heel vroeg opgestaan. | LcD_LCMI |
| 135 | De verkoopsters is vanmorgen al heel vroeg opgestaan. | LcD_LIMC |
| 136 | De verkoopster zijn vanmorgen al heel vroeg opgestaan. | LcD_LIMI |
| 137 | Daar staat de diplomate die bij het vliegtuigongeluk overleden is. | LDD_LCMC |
| 138 | Daar staan de diplomaten die bij het vliegtuigongeluk overleden zijn. | LDD_LCMI |
| 139 | Daar staat de diplomate die bij het vliegtuigongeluk overleden zijn. | LDD_LIMC |
| 140 | Daar staan de diplomaten die bij het vliegtuigongeluk overleden is. | LDD_LIMI |
| 141 | De diplomate is bij het vliegtuigongeluk overleden. | LcD_LCMC |
| 142 | De diplomaten zijn bij het vliegtuigongeluk overleden. | LcD_LCMI |
| 143 | De diplomate zijn bij het vliegtuigongeluk overleden. | LcD_LIMC |
| 144 | De diplomaten is bij het vliegtuigongeluk overleden. | LcD_LIMI |
| 145 | Daar komen de nichten die op wereldreis vertrokken zijn. | LDD_LCMC |
| 146 | Daar komt de nicht die op wereldreis vertrokken is. | LDD_LCMI |
| 147 | Daar komen de nichten die op wereldreis vertrokken is. | LDD_LIMC |
| 148 | Daar komt de nicht die op wereldreis vertrokken zijn. | LDD_LIMI |
| 149 | De nichten zijn op wereldreis vertrokken. | LcD_LCMC |
| 150 | De nicht is op wereldreis vertrokken. | LcD_LCMI |
| 151 | De  nichten is op wereldreis vertrokken. | LcD_LIMC |
| 152 | De  nicht zijn op wereldreis vertrokken. | LcD_LIMI |
| 153 | Daar staat de domme neef die de hele familie misgelopen is. | LDD_LCMC |
| 154 | Daar staan de domme neven die de hele familie misgelopen zijn. | LDD_LCMI |
| 155 | Daar staat de domme neef die de hele familie misgelopen zijn. | LDD_LIMC |
| 156 | Daar staan de domme neven die de hele familie misgelopen is. | LDD_LIMI |
| 157 | De domme neef is de hele familie misgelopen. | LcD_LCMC |
| 158 | De domme neven zijn de hele familie misgelopen. | LcD_LCMI |

| | | |
|---|---|---|
| 159 | De domme neef zijn de hele familie misgelopen. | LcD_LIMC |
| 160 | De domme neven is de hele familie misgelopen. | LcD_LIMI |
| 161 | Daar komen de peetooms die door de grachten gezwommen zijn. | LDD_LCMC |
| 162 | Daar komt de peetoom die door de grachten gezwommen is. | LDD_LCMI |
| 163 | Daar komen de peetooms die door de grachten gezwommen is. | LDD_LIMC |
| 164 | Daar komt de peetoom die door de grachten gezwommen zijn. | LDD_LIMI |
| 165 | De peetooms zijn door de  grachten gezwommen. | LcD_LCMC |
| 166 | De peetoom is door de  grachten gezwommen. | LcD_LCMI |
| 167 | De peetooms is door de  grachten gezwommen. | LcD_LIMC |
| 168 | De peetoom zijn door de  grachten gezwommen. | LcD_LIMI |
| 169 | Daar staat de slanke tante die door een streng dieet afgevallen is. | LDD_LCMC |
| 170 | Daar staan de slanke tantes die door een streng dieet afgevallen zijn. | LDD_LCMI |
| 171 | Daar staat de slanke tante die door een streng en nieuw dieet afgevallen zijn. | LDD_LIMC |
| 172 | Daar staan de slanke tantes die door een streng en nieuw dieet afgevallen is. | LDD_LIMI |
| 173 | De slanke tante is door een streng dieet afgevallen. | LcD_LCMC |
| 174 | De slanke tantes zijn door een streng dieet afgevallen. | LcD_LCMI |
| 175 | De slanke tante zijn door een streng en nieuw dieet afgevallen. | LcD_LIMC |
| 176 | De slanke tantes is door een streng en nieuw dieet afgevallen. | LcD_LIMI |
| 177 | Daar komen de moedige matrozen die over de woeste zee gevaren zijn. | LDD_LCMC |
| 178 | Daar komt de moedige matroos die over de woeste zee gevaren is. | LDD_LCMI |
| 179 | Daar komen de moedige matrozen die over de erg woeste zee gevaren is. | LDD_LIMC |
| 180 | Daar komt de moedige matroos die over de erg woeste zee gevaren zijn. | LDD_LIMI |
| 181 | De moedige matrozen zijn over de woeste zee gevaren. | LcD_LCMC |
| 182 | De moedige matroos is over de woeste zee gevaren. | LcD_LCMI |
| 183 | De moedige matrozen is over de erg woeste zee gevaren. | LcD_LIMC |
| 184 | De moedige matroos zijn over de erg woeste zee gevaren. | LcD_LIMI |
| 185 | Daar staat de bazin die door een blunder afgetreden is. | LDD_LCMC |
| 186 | Daar staan de bazinnen die door een blunder afgetreden zijn. | LDD_LCMI |
| 187 | Daar staat de bazin die door een blunder afgetreden zijn. | LDD_LIMC |
| 188 | Daar staan de bazinnen die door een blunder afgetreden is. | LDD_LIMI |
| 189 | De bazin is door een blunder afgetreden. | LcD_LCMC |
| 190 | De bazinnen zijn door een blunder afgetreden. | LcD_LCMI |
| 191 | De bazin zijn door een blunder afgetreden. | LcD_LIMC |
| 192 | De bazinnen is door een blunder afgetreden. | LcD_LIMI |
| 193 | Daar komen de vaders die vroeger stout op school geweest zijn. | LDD_LCMC |
| 194 | Daar komt de vader die vroeger stout op school geweest is. | LDD_LCMI |
| 195 | Daar komen de vaders die vroeger stout op school geweest is. | LDD_LIMC |
| 196 | Daar komt de vader die vroeger stout op school geweest zijn. | LDD_LIMI |
| 197 | De vaders zijn vroeger stout op school geweest. | LcD_LCMC |
| 198 | De vader is vroeger stout op school geweest. | LcD_LCMI |

| | | |
|---|---|---|
| 199 | De vaders is vroeger stout op school geweest. | LcD_LIMC |
| 200 | De vader zijn vroeger stout op school geweest. | LcD_LIMI |
| 201 | Daar staat de kitten die in de hoge bomen geklommen is. | LDD_LCMC |
| 202 | Daar staan de kittens die in de hoge bomen geklommen zijn. | LDD_LCMI |
| 203 | Daar staat de kitten die in de hoge bomen geklommen zijn. | LDD_LIMC |
| 204 | Daar staan de kittens die in de hoge bomen geklommen is. | LDD_LIMI |
| 205 | De kitten is in de hoge bomen geklommen. | LcD_LCMC |
| 206 | De kittens zijn in de hoge bomen geklommen. | LcD_LCMI |
| 207 | De kitten zijn in de hoge bomen geklommen. | LcD_LIMC |
| 208 | De kittens is in de hoge bomen geklommen. | LcD_LIMI |
| 209 | Daar komen de getrainde honden die over het slootje gesprongen zijn. | LDD_LCMC |
| 210 | Daar komt de getrainde hond die over het slootje gesprongen is. | LDD_LCMI |
| 211 | Daar komen de getrainde honden die over het slootje gesprongen is. | LDD_LIMC |
| 212 | Daar komt de getrainde hond die over het slootje gesprongen zijn. | LDD_LIMI |
| 213 | De getrainde honden zijn over het slootje gesprongen. | LcD_LCMC |
| 214 | De getrainde hond is over het slootje gesprongen. | LcD_LCMI |
| 215 | De getrainde honden is over het slootje gesprongen. | LcD_LIMC |
| 216 | De getrainde hond zijn over het slootje gesprongen. | LcD_LIMI |
| 217 | Daar staat de inwoonster die naar het buurland gevlucht is. | LDD_LCMC |
| 218 | Daar staan de inwoonsters die naar het buurland gevlucht zijn. | LDD_LCMI |
| 219 | Daar staat de inwoonster die naar het buurland gevlucht zijn. | LDD_LIMC |
| 220 | Daar staan de inwoonsters die naar het buurland gevlucht is. | LDD_LIMI |
| 221 | De inwoonster is naar het buurland gevlucht. | LcD_LCMC |
| 222 | De inwoonsters zijn naar het buurland gevlucht. | LcD_LCMI |
| 223 | De inwoonsters zijn naar het buurland gevlucht. | LcD_LIMC |
| 224 | De inwoosnters is naar het buurland gevlucht. | LcD_LIMI |
| 225 | Daar komen de slordige klanten die alle bonnetjes kwijtgeraakt zijn. | LDD_LCMC |
| 226 | Daar komt de slordige klant die alle bonnetjes kwijtgeraakt is. | LDD_LCMI |
| 227 | Daar komen de slordige klanten die alle bonnetjes kwijtgeraakt is. | LDD_LIMC |
| 228 | Daar komt de slordige klant die alle bonnetjes kwijtgeraakt zijn. | LDD_LIMI |
| 229 | De slordige klanten zijn alle bonnetjes kwijtgeraakt. | LcD_LCMC |
| 230 | De slordige klant is alle bonnetjes kwijtgeraakt. | LcD_LCMI |
| 231 | De slordige klanten is alle bonnetjes kwijtgeraakt. | LcD_LIMC |
| 232 | De slordige klant zijn alle bonnetjes kwijtgeraakt. | LcD_LIMI |
| 233 | Daar staat de beschermende leidster die tegen het grote onrecht opgetreden is. | LDD_LCMC |
| 234 | Daar staan de beschermende leidsters die tegen het grote onrecht opgetreden zijn. | LDD_LCMI |
| 235 | Daar staat de beschermende leidster die tegen het grote onrecht opgetreden zijn. | LDD_LIMC |
| 236 | Daar staan de beschermende leidsters die tegen het grote onrecht opgetreden is. | LDD_LIMI |
| 237 | De beschermende leidster is tegen het grote onrecht opgetreden. | LcD_LCMC |
| 238 | De beschermende leidsters zijn tegen het grote onrecht opgetreden. | LcD_LCMI |

| | | |
|---|---|---|
| 239 | De beschermende leidster zijn tegen het onrecht opgetreden. | LcD_LIMC |
| 240 | De beschermende leidsters is tegen het grote onrecht opgetreden. | LcD_LIMI |
| 241 | Daar komen de kelners die in het chique restaurant beland zijn. | LDD_LCMC |
| 242 | Daar komt de kelner die in het chique restaurant beland is. | LDD_LCMI |
| 243 | Daar komen de kelners die in het chique restaurant beland is. | LDD_LIMC |
| 244 | Daar komt de kelner die in het chique restaurant beland zijn. | LDD_LIMI |
| 245 | De kelners zijn in het chique restaurant beland. | LcD_LCMC |
| 246 | De kelner is in het chique restaurant beland. | LcD_LCMI |
| 247 | De kelners is in het chique restaurant beland. | LcD_LIMC |
| 248 | De kelner zijn in het chique restaurant beland. | LcD_LIMI |
| 249 | Daar staat de nieuwe medewerker die onder de heftige druk bezweken is. | LDD_LCMC |
| 250 | Daar staan de nieuwe medewerkers die onder de heftige druk bezweken zijn. | LDD_LCMI |
| 251 | Daar saat de nieuwe medewerker die onder de heftige druk bezweken zijn. | LDD_LIMC |
| 252 | Daar staan de nieuwe medewerkers die onder de heftige druk bezweken is. | LDD_LIMI |
| 253 | De nieuwe medewerker is onder de heftige druk bezweken. | LcD_LCMC |
| 254 | De nieuwe medewerkers zijn onder de heftige druk bezweken. | LcD_LCMI |
| 255 | De nieuwe medewerker zijn onder de heftige druk bezweken. | LcD_LIMC |
| 256 | De nieuwe medewerkers is onder de heftige druk bezweken. | LcD_LIMI |
| 257 | Daar komen de voorzichtige voetgangers die de drukke straat overgestoken zijn. | LDD_LCMC |
| 258 | Daar komt de voorzichtige voetganger die de drukke straat overgestoken is. | LDD_LCMI |
| 259 | Daar komen de voorzichtige voetgangers die de drukke straat overgestoken is. | LDD_LIMC |
| 260 | Daar komt de voorzichtige voetganger die de drukke straat overgestoken zijn. | LDD_LIMI |
| 261 | De voorzichtige voetgangers zijn de drukke straat overgestoken. | LcD_LCMC |
| 262 | De voorzichtige voetganger is de drukke straat overgestoken. | LcD_LCMI |
| 263 | De voorzichtige voetgangers is de drukke straat overgestoken. | LcD_LIMC |
| 264 | De voorzichtige voetganger zijn de drukke straat overgestoken. | LcD_LIMI |
| 265 | Daar staat de warrige monteur die enkele schroeven verloren is. | LDD_LCMC |
| 266 | Daar staan de warrige monteurs die enkele schroeven verloren zijn. | LDD_LCMI |
| 267 | Daar staat de warrige monteur die enkele schroeven verloren zijn. | LDD_LIMC |
| 268 | Daar staan de warrige monteurs die enkele schroeven verloren is. | LDD_LIMI |
| 269 | De warrige monteur is enkele schroeven verloren. | LcD_LCMC |
| 270 | De warrige monteurs zijn enkele schroeven verloren. | LcD_LCMI |
| 271 | De warrige monteur zijn enkele schroeven verloren. | LcD_LIMC |
| 272 | De warrige monteurs is enkele schroeven verloren. | LcD_LIMI |
| 273 | Daar komen de kwajongens die van de glijbaan gegleden zijn. | LDD_LCMC |
| 274 | Daar komt de kwajongen die van de glijbaan gegleden is. | LDD_LCMI |
| 275 | Daar komen de kwajongens die van de glijbaan gegleden is. | LDD_LIMC |
| 276 | Daar komt de kwajongen die van de glijbaan gegleden zijn. | LDD_LIMI |
| 277 | De kwajongens zijn van de glijbaan gegleden. | LcD_LCMC |
| 278 | De kwajongen is van de glijbaan gegleden. | LcD_LCMI |

| | | |
|---|---|---|
| 279 | De kwajongens is van de glijbaan gegleden. | LcD_LIMC |
| 280 | De kwajongen zijn van de glijbaan gegleden. | LcD_LIMI |
| 281 | Daar staat de leraar die na de les langer gebleven is. | LDD_LCMC |
| 282 | Daar staan de leraren die na de les langer gebleven zijn. | LDD_LCMI |
| 283 | Daar staat de leraar die na de les langer gebleven zijn. | LDD_LIMC |
| 284 | Daar staan de leraren die na de les langer gebleven is. | LDD_LIMI |
| 285 | De leraar is na de les langer gebleven. | LcD_LCMC |
| 286 | De leraren zijn na de les langer gebleven. | LcD_LCMI |
| 287 | De leraar zijn na de les langer gebleven. | LcD_LIMC |
| 288 | De leraren is na de les langer gebleven. | LcD_LIMI |

LDD = Long-distance dependency
LcD = Local dependency

LCMC = language correct, music correct
LIMC = Language incorrect, music correct
LCMI = Language correct, music incorrect
LIMI = Language incorrect, music correct
